# The Clinical Prediction Value of the Ubiquitination Model Reflecting the Immune Traits in LUAD

**DOI:** 10.3389/fimmu.2022.846402

**Published:** 2022-02-25

**Authors:** Yinggang Che, Dongbo Jiang, Leidi Xu, Yuanjie Sun, Yingtong Wu, Yang Liu, Ning Chang, Jiangjiang Fan, Hangtian Xi, Dan Qiu, Qing Ju, Jingyu Pan, Yong Zhang, Kun Yang, Jian Zhang

**Affiliations:** ^1^ Department of Pulmonary and Critical Care Medicine, Xijing Hospital, Air-Force Medical University, Xi’an, China; ^2^ Department of Immunology, Basic Medicine School, Air-Force Medical University, Xi’an, China; ^3^ Department of First Sanatorium, First Sanatorium of Air Force Healthcare Center for Special Services, Hangzhou, China; ^4^ Shaanxi Provincial Center for Disease Control and Prevention, Xi’an, China; ^5^ Department for AIDS Prevention and Control, Department of Thoracic Surgery, Tangdu Hospital, Air-Force Medical University, Xi’an, China

**Keywords:** ubiquitination, prognostic model, immune infiltration, genome mutation, drug response

## Abstract

**Background:**

Increasing evidence shows that the ubiquitin–proteasome system has a crucial impact on lung adenocarcinoma. However, reliable prognostic signatures based on ubiquitination and immune traits have not yet been established.

**Methods:**

Bioinformatics was performed to analyze the characteristic of ubiquitination in lung adenocarcinoma. Principal component analysis was employed to identify the difference between lung adenocarcinoma and adjacent tissue. The ubiquitin prognostic risk model was constructed by multivariate Cox regression and least absolute shrinkage and selection operator regression based on the public database The Cancer Genome Atlas, with evaluation of the time-dependent receiver operating characteristic curve. A variety of algorithms was used to analyze the immune traits of model stratification. Meanwhile, the drug response sensitivity for subgroups was predicted by the “pRRophetic” package based on the database of the Cancer Genome Project.

**Results:**

The expression of ubiquitin genes was different in the tumor and in the adjacent tissue. The ubiquitin model was superior to the clinical indexes, and four validation datasets verified the prognostic effect. Additionally, the stratification of the model reflected distinct immune landscapes and mutation traits. The low-risk group was infiltrating plenty of immune cells and highly expressed major histocompatibility complex and immune genes, which illustrated that these patients could benefit from immune treatment. The high-risk group showed higher mutation and tumor mutation burden. Integrating the tumor mutation burden and the immune score revealed the patient’s discrepancy between survival and drug response. Finally, we discovered that the drug targeting ubiquitin and proteasome would be a beneficial prospective treatment for lung adenocarcinoma.

**Conclusion:**

The ubiquitin trait could reflect the prognosis of lung adenocarcinoma, and it might shed light on the development of novel ubiquitin biomarkers and targeted therapy for lung adenocarcinoma.

## Introduction

It has been extensively acknowledged that lung cancer is strikingly the most common cancer among the whole population (11.6% of the total cases) and the leading cause of cancer death (18.4% of the total cancer deaths). Lung adenocarcinoma (LUAD), the predominant subtype of non-small cell lung cancer, features a poor prognosis and a limited 5-year survival rate (1, 2). However, patients diagnosed with advanced LUAD, specifically those who fail to take surgical interventions, are liable to suffer from retardant clinical diagnosis and inadequate treatment regimes, which, in turn, lead to a worsened status with restricted survival. It is of necessity to regard risk assessment as a priority to detect those in early stages and take further radical measures aimed to prevent progression.

Ubiquitination, a frequent post-translational modification that is highly conserved for metazoans and regulates the stability and degradation of proteins, usually functions reversibly within a series of enzyme-dependent reactions (3). It has the potency to modify tumor-associated proteins and further degrade them in a proteasome-dependent manner that makes the malfunction of ubiquitination an adverse capacity to cause LUAD inclusively (4–9). There still exists a necessity to further uncover the diagnostic and prognostic value of ubiquitin–proteasome systems in LUAD. Interestingly, several recent studies indicated that ubiquitination serves as a crucial adaptor in the regulation of innate and adaptive immune responses as well as immune tolerance (10). Being proven markers of dendritic cell maturation, MHC class II (MHCII) and costimulatory molecules on the cell membranes, such as CD80 and CD86, are regulated by ubiquitination–deubiquitylation-dependent dynamic equilibrium (11). Similarly, ubiquitination also correlates with the regulation of T cell receptor proximal signaling, which acts as a critical component of adaptive immunity. These results indicated that ubiquitination is involved in extensive antitumor immunity but failed to describe its explicit role in regulating immune cells and their environment. Thus, the exploration of ubiquitination in regulating immune response and its correlation with genome alternation in lung adenocarcinoma needs further evaluation.

In this study, we found that the ubiquitin molecules were different in the tumor and in the adjacent tissue and further observed the potential biological traits at the transcriptome and protein levels. Subsequently, we constructed a ubiquitination-oriented predictive model on the basis of a public database analysis to evaluate its ubiquitin degree and prognostic value in LUAD. Using integrated and stratified multi-omics analysis within immune infiltration and genome alternation, respectively, we further explored its clinical efficacy in predicting prognosis and drug response to immune checkpoint blockade and targeted therapy with the present evaluative markers. Overall, our study presented a brand new clinically predictive ubiquitin model, which aims to uncover the underlying ubiquitination characteristics of LUAD and its clinical predictive effectiveness with different genotypes.

## Methods

### Principal Component Analysis of Ubiquitin-Associated Genes in LUAD and Adjacent Tissue

In total, 2,838 ubiquitination genes were integrated, which originated from the Integrated Annotations for Ubiquitin and Ubiquitin-Like Conjugation Database (IUUCD) (http://iuucd.biocuckoo.org/) (12). We found that 181 ubiquitin genes were co-expressed in LUAD. The principal component analysis of the 181 ubiquitin genes screened revealed the different expression in the tumors and the adjacent tissues. It was performed by the “pca3d” packages of R studio and visualized.

### Construction of the Prognostic Risk Model

Cox proportional hazard regression was used to evaluate the prognosis-related ubiquitin genes in the expression of 181 ubiquitination genes in patients with LUAD. A total of 26 differentially expressed genes in 181 genes were associated with the prognosis of LUAD patients. Subsequently, 10 among the 26 prognostic genes were screened to construct the multivariate Cox model using stepwise multivariate Cox proportional hazard regression (*p* < 0.05). Meanwhile, 11 of the 26 genes were selected to build the prognostic model by least absolute shrinkage and selection operator (LASSO) regression. Then, the two models intersected with 9 genes: USP29, MPP7, TRIM40, HERC1, TLE1, ASB2, NEDD1, USP44, and PHF1, respectively. LASSO Cox regression was performed to reconstruct the prognostic model of 9 genes.

A ubiquitin-related risk score (URS) was established by including the gene expression values weighted by Cox multivariate proportional risk model coefficients:

URS = ∑_i_[coefficient (mRNA_i_) × expression (mRNA_i_)]. The three models were as follows: multivariate Cox model—URS_multi-Cox_ =USP29 * 0.35 + MPP7 * (-0.23) + TRIM4 * 0.08 + HERC1 * (-0.28) + TLE1 * 0.36 + RNF166 * 0.25 + ASB2 * (-0.29) + NEDD1 * 0.48 + USP44 * (-0.1) + PHF1 * (-0.36); LASSO model of 11 genes—URS_LASSO (11)_ = USP29 * 0.28 + MPP7 * (-0.18) + TLE2 * (-0.04) + TRIM40 * 0.04 + HERC1 * (-0.16) + TLE1 * 0.32 + ASB2 * (-0.2) + NEDD1 * 0.32+USP44 * (-0.8) +PHF1 * (-0.15) + WSB2 * 0.03; and LASSO model of 9 genes—URS_LASSO (9)_ = USP29 * 0.34 + MPP7 * (-0.2) + TRIM40 * 0.06 + HERC1 * (-0.21) + TLE1 * 0.36 + ASB2 * (-0.2) + NEDD1 * 0.41 + USP44 * (-0.1) + PHF1 * (-0.22)

In addition, overall survival and first progression of each gene of the URS_LASSO (9)_ model were analyzed by the Kaplan–Meier (KM) plotter (http://kmplot.com/analysis/).

### Validation of the Model

According to the pathology stage of the patients, they were spilt into the early and advanced stages. Stages I and II were considered as the early stage, and stages III and IV were assigned to the advanced stage of LUAD patients, including 378 and 104 patients, respectively.

The outer four validation datasets verified the stability and the accuracy of the prognostic risk model from the Gene Expression Omnibus (GEO) datasets, including GSE13213, GSE31210, GSE36471, and GSE11969.

### Analysis With PPI and GSEA of the Model

The functional protein interaction network of the 9 ubiquitin genes was predicted using the STRING database (https://stringb.org/) and considering the interacting protein based on the interaction score >0.70. A total of 71 molecule proteins met the screening criteria, and a protein interaction network map was constructed by using Cytoscape 3.6.1.

The genes of the enrichment pathway were analyzed using Gene Set Enrichment Analysis (GSEA) based on Gene Ontology (GO) and Kyoto Encyclopedia of Genes and Genomes (KEGG). Three KEGG pathways were co-expressed corresponding to each of the 9 Ubi genes.

### Immune Infiltration of the Prognostic Risk Model

According to the prognostic risk score, the patients of LUAD of TCGA databases were split into high- and low-risk groups. The differentially expressed genes of the groups was determined by the LIMMA package (13) of R software, and the genes were selected by |log fold change| ≥2 and *p*-value ≤10^-5^.

The levels of infiltrating immune and stromal cells were calculated by QUANTISEQ, CIBERSORT, and XCELL algorithms, which included 22 cells of the CIBERSORT algorithm, 11 cells of the QUANTISEQ algorithm, and 64 cells of the XCELL algorithm. The immune score, stroma score, and microenvironment score were calculated by the XCELL algorithms. The t-distributed stochastic neighbor embedding (t-SNE) was used to analyze the clusters of immune cells of the three algorithms to dimensionality reduction.

### Mutation Profile of the Model

According to the model grouping, there were 242 cases in the high-risk group and 244 cases in the low-risk group. The mutation data of LUAD was analyzed and visualized by the maftools package of R software. The co-mutation of pair genes was calculated by somatic interaction function and examined by Fisher’s exact test, and the tumor mutation burden (TMB) is derived as mutation number/30. The patients were divided into four subgroups according to the mean of TMB and immune scores, such as high TMB and low immune score, high TMB and high immune score, low TMB and low immune score, and low TMB and high immune score, respectively.

### Clinical Drug Response

Based on a database of the Cancer Genome Project (CGP), we screened 4 chemotherapy drugs (cisplatin, gemcitabine, paclitaxel, and etoposide), 3 targeted drugs (axitinib, selumetinib, and gefitinib), and 3 proteasome inhibitors (bortezomib, lenalidomide, and MG132). The “pRRophetic” package was used to analyze the drug response of chemotherapy and targeted therapy for groups, including the risk group, and a group of TMB/immune score.

### Survival and Other Statistical Analysis

For the categorical variables, the KM plotter and Cox regression analysis were used to calculate the significance of overall survival (OS). The statistical difference of the OS in the KM curve analyses was compared using the log-rank test. For continuous variables, Cox regression was used to calculate the hazard ratio and significance of differences in the OS. The time-dependent area under the receiver operating characteristic (ROC) curve (AUC) was used to evaluate the predictive power of risk score and clinical indexes to model.

The statistical difference of distribution of three or more groups was examined by the Kruskal–Wallis test and that of two groups was compared by the Wilcoxon test. Chi-square was used to examine the statistical differences of risk groups and other clinical indexes, including age, gender, stage, T stage, N stage, M stage, risk score, and risk score plus stage. The *P*-values are two-sided, and *P <*0.05 was considered statistically significant.

## Results

### Construction of the Prognostic Risk Model of Ubiquitination

**Figure 1:**
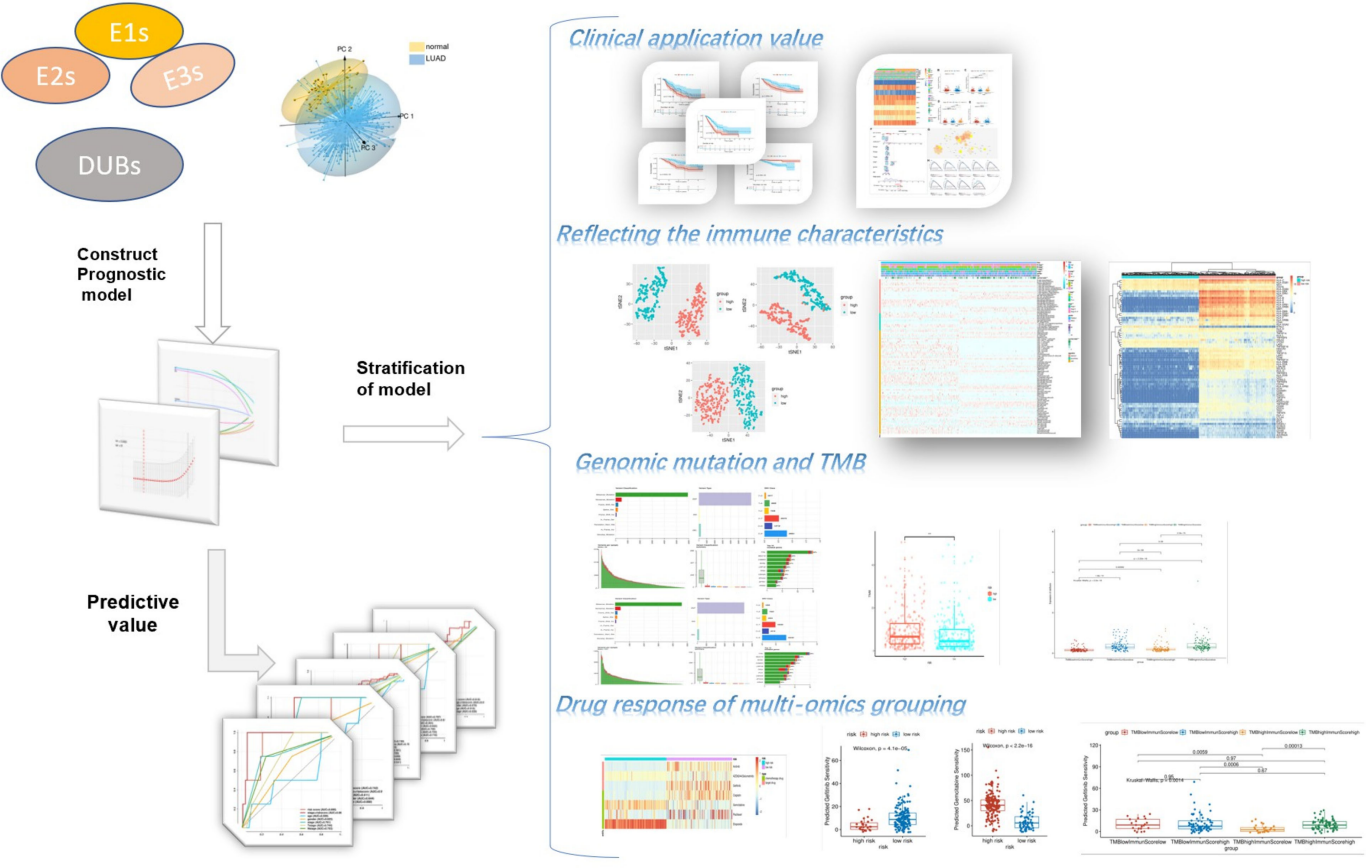
Flow chart of the research.

**Figure 2:**
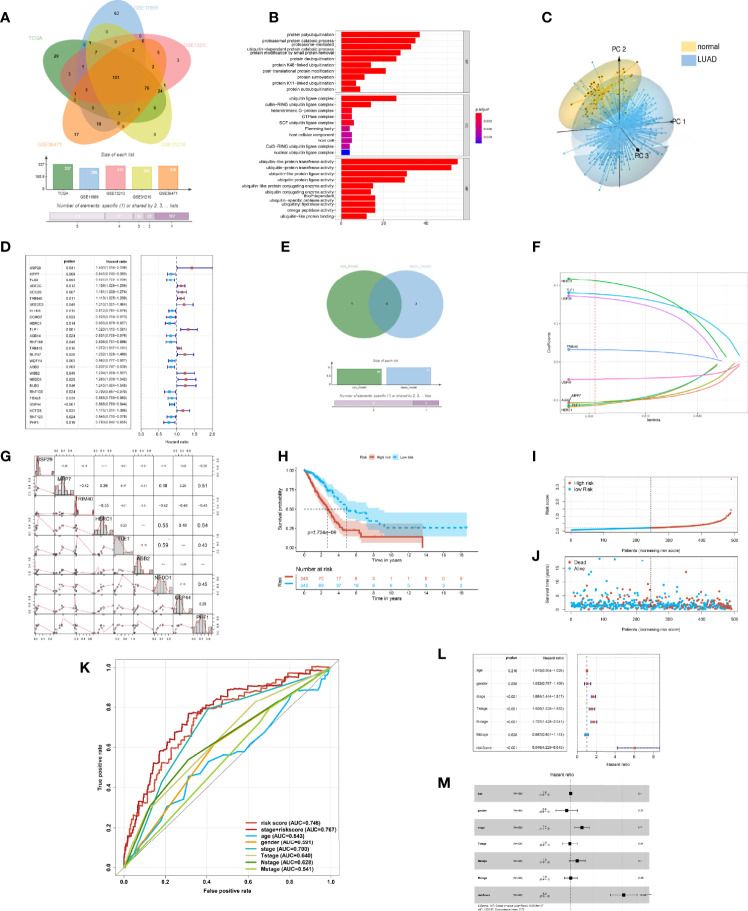
Construction of the prognostic risk model of ubiquitination in the lung adenocarcinoma (LUAD). **(A)** Ubiquitination-associated genes co-expressed in The Cancer Genome Atlas (TCGA), GSE13213, GSE31210, GSE36471, and GSE11969 datasets. **(B)** Bar plot of the Gene Ontology enrichment analysis of ubiquitin genes. **(C)** 3D principal component analysis plot of ubiquitin genes in LUAD and adjacent tissue. **(D)** Statistically significant (*p* < 0.05) Ubi genes of the prognostic model based on univariate Cox proportion hazards regression. **(E)** Venn plot intersecting genes of the LASSO model and multivariate Cox model. **(F)** LASSO plot of 9 genes with ubiquitination LASSO model. **(G)** Correlation of the Ubi genes of the prognostic risk model. **(H)** Kaplan–Meier curve of the high- and low-risk groups of TCGA-LUAD. **(I)** Risk score of the LASSO prognostic model. **(J)** Risk state of the LASSO prognostic model. **(K)** Receiver operating characteristic curve of the risk score and other clinical factors for TCGA-LUAD. **(L)** Forest plot of the univariate Cox regression analysis of the risk score and clinical factors for TCGA-LUAD. **(M)** Forest plot of the multivariate Cox regression analysis of the risk score and clinical factors for TCGA-LUAD.

The ubiquitin–proteasome system was a signature pathway to hydrolytic protease and participated in the process of lung adenosarcoma. To research the signature of ubiquitin molecules in patients of LUAD, 2,838 ubiquitination genes were screened, including those encoding E1s (ubiquitin-activating enzymes), E2s (ubiquitin-conjugating enzymes), E3s (ubiquitin-protein ligases), and DUBs (deubiquitinating enzymes), which originated from the IUUCD. Our study flow chart is illustrated in **Figure 1**. A total of 181 genes of ubiquitination were co-expressed in the TCGA and GEO databases (**Figure 2A**). The demographics of this cohort are listed in **Table 1**. The GO pathway enrichment analysis was performed to uncover whether these ubiquitination genes were involved in the protein ubiquitination-related biological process (**Figure 2B**). The genes were assigned to two clusters of LUAD and adjacent tissue by principal component analysis, which revealed that the ubiquitin genes influenced the biological process of LUAD and needed further research (**Figure 2C**).

**Table 1:** Clinical characteristics of the patients from multiple datasets.

	TCGA (*n* = 522)	GSE11969 (*n* = 90)	GSE13213 (*n* = 117)	GES31210 (*n* = 226)	GSE36471 (*n* = 116)
Age, years					
Median	65.3	61	60.7	59.6	60
NA	18 (3.4%)	–	–	–	–
Gender					
Male	242 (46.4%)	47 (52.2%)	60 (51.3%)	105 (46.5%)	53 (45.7%)
Female	280 (53.6%)	43 (47.8%)	57 (48.7%)	121 (53.5%)	63 (54.3%)
TNM stage					
Stage I	279 (53.4%)	40 (44.4%)	54 (46.2%)	168 (74.3%)	62 (53.4%)
Stage II	124 (23.8%)	37 (41.1%)	50 (42.7%)	58 (25.7%)	19 (16.4%)
Stage III	85 (16.3%)	8 (8.9%)	8 (6.8%)	–	19 (16.4%)
Stage IV	26 (5.0%)	5 (5.6%)	5 (4.3%)	–	1 (0.9%)
NA	8 (1.5%)	–	–	–	15 (12.9%)
OS state					
Alive	355 (68.0%)	50 (55.6%)	68	191 (84.5%)	49 (42.2%)
Dead	167 (32.0%)	40 (44.4%)	49	35 (15.5%)	66 (56.9%)
NA	–	–	–	–	1 (0.9%)

Data are presented as n (%). NA, not available; OS, overall survival.

In order to reflect the ubiquitin level and clinical prognosis of LUAD, we attempted to construct the evaluation criteria. Therefore, by Cox regression analysis, 26 ubiquitin genes of co-expressing multidatasets were associated with the prognosis of patients with LUAD (*p* < 0.05) (**Figure 2D**). To illuminate the ubiquitin characteristics, we constructed three models of ubiquitin gene signature patients prognosis by different methods. In these genes, 10 genes were screened to build the URS_multi-cox_ of the prognostic model using stepwise multivariate Cox proportional hazard regression. Then, 11 genes were equally selected to build the URS_LASSO (11)_ of the prognostic model by LASSO regression. By intersecting the crucial genes of URS_multi-cox_ and URS_LASSO (11)_ models, 9 ubiquitin genes of URS_LASSO (9)_ model were constructed by LASSO regression (**Figure 2E**).

Upon comparing the three prognostic risk models, there were no differences found in OS (*p* < 0.001) (**Supplementary Figures S1A, C, E**). Simultaneously, there was a little difference for the AUC of time-dependent ROC on the three prognostic risk models. The 1-year AUC of URS_multi-cox_, URS_LASSO (11)_, and URS_LASSO (9)_ were 0.740, 0.745, and 0.746, respectively (**Supplementary Figures S1B, D, F**). It revealed that 9 ubiquitin genes played a crucial role in lung adenocarcinoma, including USP29, MPP7, TRIM40, HERC1, TLE1, ASB2, NEDD1, USP44, and PHF1. Therefore, it was reasonable to consider that the URS_LASSO (9)_ model predicted the prognostic risk of LUAD patients and reflected the ubiquitin signature (**Figure 2F**). Additionally, each gene of the model had critical importance in the overall survival and first progression, which would turn into the new prognostic biomarkers of LUAD (**Supplementary Figures S2**, **S3**). PHF1 and MPP7 had a high correlation according to the transcriptome data (*r* = 0.59) (**Figure 2G**).

For the prognostic risk model, the patients of the high-score group had a poor prognosis (**Figure 2H**). There was a higher risk score for the high-risk model, which shows that patients with increasing scores accumulated their risks. With increasing risk score, the death toll was equally higher and the survival time was shorter (**Figures 2I, J**). Notably, the ROC of the pathology stage plus risk score was better than the other indexes, such as risk score, age, gender, and TMN stage (**Figure 2K**). In the analysis of Cox regression between risk score and clinical indexes, the model would predict the prognostic risk as an independent factor (**Figures 2L, M**). It illustrated that the model would more comprehensively evaluate and predict a patient’s risk as a complementary method. The potential value of the model for predicting the prognosis of patients and in assisting diagnosis was likewise demonstrated. As a result, the ubiquitin–proteasome system was a crucial signature in patients with LUAD.

### Reliability of the Model in Early and Advanced LUAD Patients and Validation in the Four Independent LUAD Cohorts

**Figure 3:**
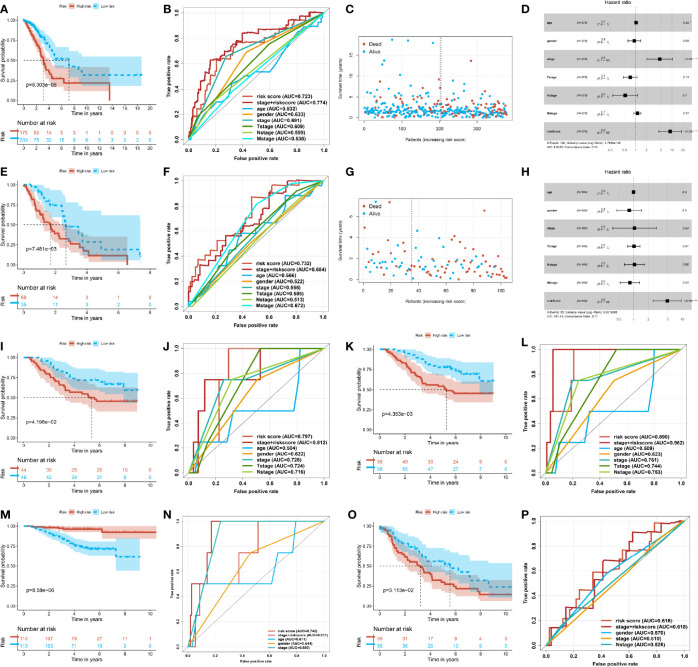
Stability and predictive power of the prognostic risk model. **(A)** Kaplan–Meier curve of the high- and low-risk group in the early lung adenocarcinoma (LUAD) of The Cancer Genome Atlas (TCGA). **(B)** Receiver operating characteristic (ROC) curve of the risk score and other clinical indexes in the early LUAD of TCGA. **(C)** Scatter plot of the survival time and risk score in the early LUAD of TCGA. **(D)** Forest plot of multivariate Cox regression analysis of the risk score and clinical factors for the early LUAD of TCGA. **(E)** Kaplan–Meier curve of the high- and low-risk groups in the progressive LUAD of TCGA. **(F)** ROC curve of the risk score and other clinical indexes in the progressive LUAD of TCGA. **(G)** Scatter plot of the survival time and risk score in the progressive LUAD of TCGA. **(H)** Forest plot of the multivariate Cox regression analysis of the risk score and clinical factors for the progressive LUAD of TCGA. **(I)** Kaplan–Meier curve of the high- and low-risk groups of GSE11969. **(J)** ROC curve of the risk score and other clinical factors from GSE11969. **(K)** Kaplan–Meier curve of the high- and low-risk groups of patients from GSE13213. **(L)** ROC curve of the risk score and other clinical factors of GSE13213. **(M)** Kaplan–Meier curve of the high- and low-risk groups of patients from GSE31210. **(N)** ROC curve of the risk score and other clinical factors of GSE31210. **(O)** Kaplan–Meier curve of the high- and low-risk groups of patients from GSE36471. **(P)** ROC curve of the risk score and other clinical factors of patients from GSE36471.

In order to examine the feasibility and the reliability of the prognostic model, we divided the clinical stage into the early stage and the progressive stage. The patients were classified into early (stages I and II) and advanced groups (stages III and IV), covering 378 cases and 104 cases, respectively. For early-stage LUAD patients, the low-score group had a more favorable OS, and the number of patients in the low-risk group was higher than in the high-risk group (**Figure 3A**). The ROC of the risk score preceded the other clinical indexes (**Figure 3B**). As for lower risk scores, the survival time of patients was longer, and the number of patients was even more (**Figure 3C**). Accordingly, the risk model was applied to the early LUAD and reflected the patient’s prognostic as a risk factor (**Figure 3D**). Inversely, in terms of the advanced stage, the number of patients in the high-risk group was more than in the low-risk group, and there was a poorer OS in the high-risk group (**Figure 3E**). The ROC of risk score was superior to other indexes, and the high-risk score was shorter for survival time and more for death toll (**Figures 3F, G**). As an independent prognostic biomarker, the model could predict the risk of advanced patients (**Figure 3H**). These results demonstrated that the prognostic risk model could predict the risk state and was exempt from the pathology stage—that was to say, the ubiquitin model would predict the prognostic risk in the early LUAD patients, and it made an appropriate clinical decision of a surgical intervention.

The other transcriptome data of LUAD got similar analysis outcomes. The four independent datasets were examined by the prognostic value from GEO, such as GSE11969, GSE13213, GSE31210, and GSE36471. In the four independent datasets, the patients of the high-score group had a poorer prognosis. The median survival time of the high-score group was shorter than that of the low-score group (**Figures 3I, K, M, O** **)**. Meanwhile, the ROC of the risk score was superior to other single indexes. In addition, the ROC of the pathological stage plus risk score was superior to the single index (**Figures 3J, L, N, P**). The above-mentioned outcomes illustrated that the prognostic risk model, altogether with the current clinical diagnosis, could be more accurate and comprehensive to predict the risk and prognosis of LUAD patients. This approach could help make appropriate clinical decisions and surgical interventions.

### Model Was Associated With Clinical Indexes and Signal Pathways in LUAD

**Figure 4:**
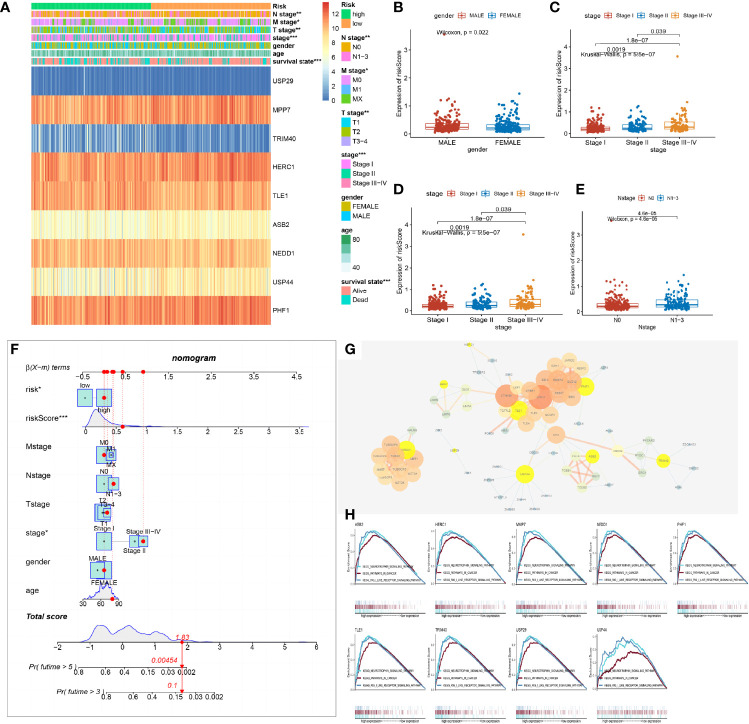
Relationship between the prognostic risk model and the clinical indexes. **(A)** Heat map of the model’s stratification, clinical indexes, and gene prognostic model. **(B–E)** Box plot showing the difference between high- and low-risk groups about gender, stage, N stage, and T stage. *P*-values were calculated with the Wilcoxon test. **(F)** Nomogram model of the risk score and other clinical factors to predict the progression of lung adenocarcinoma (LUAD). **(G)** Protein and protein network interaction of 9 model genes. Yellow color represents the 9 model genes. The size of the circle and the thickness of the line represent the combined score. **(H)** Gene Set Enrichment Analysis of a single gene of the 9 model genes associated with the low and high expression of LUAD of The Cancer Genome Atlas (TCGA) and demonstrated in the three commonly participating pathways, including neurotrophic, cancer, and Rig I-like receptor signaling pathways.

The stratification of the model demonstrated the advantage of managing LUAD patients. Upon examination using chi-square test, the stratification of the model had a potential relationship with clinical indexes (**Figure 4A**). Therefore, we explored the correlation between the risk score of the model and the clinical indexes. We found out that male patients were prone to acquire a high risk than female patients with LUAD (**Figure 4B**). Besides this, the patients of pathological stage I were at a lower risk, and with increasing on pathology stage, the risk score of the patients was increasing (**Figure 4C**). Correspondingly, with the invasion of primary sites and infiltration of lymph glands about tumor tissue, the risk of the patients was highly increasing (**Figures 4D, E**). The model could partially represent the clinical signature of patients.

As a result of the potential predictive value of the model and clinical indexes, a nomogram was constructed to speculate the probability of survival time. Adding all of the index scores together multiplies the probability of 3 or 5 years to acquire the risk of patients. The qualification of risk could improve the management of clinical patients. It helped make a clinical decision with progression in LUAD and expected to acquire beneficial OS and prognosis of patients (**Figure 4F**).

Furthermore, the 9 gene’s protein–protein interaction (PPI) of the model was built to find out the molecule’s interaction with the prognostic model. We found out that the 62 molecules were in a tight correlation with the 9 model molecules and were involved in the process of ubiquitin. The 9 genes of the model were located in the critical hub (**Figure 4G**).

Analyzing each gene by pathway enrichment of GSEA determined which pathways played a vital role in LUAD bio-progression. We discovered that the 9 genes of the model co-participated in the neurotrophy, cancer signal, and RIG-like receptor signal pathway (**Figure 4H**). Some researchers had reported that three pathways took part in LUAD bio-process by multi-ways (14–16).

### Stratification of the Model Reflected the Immune Cell and Microenvironment Characteristics

**Figure 5:**
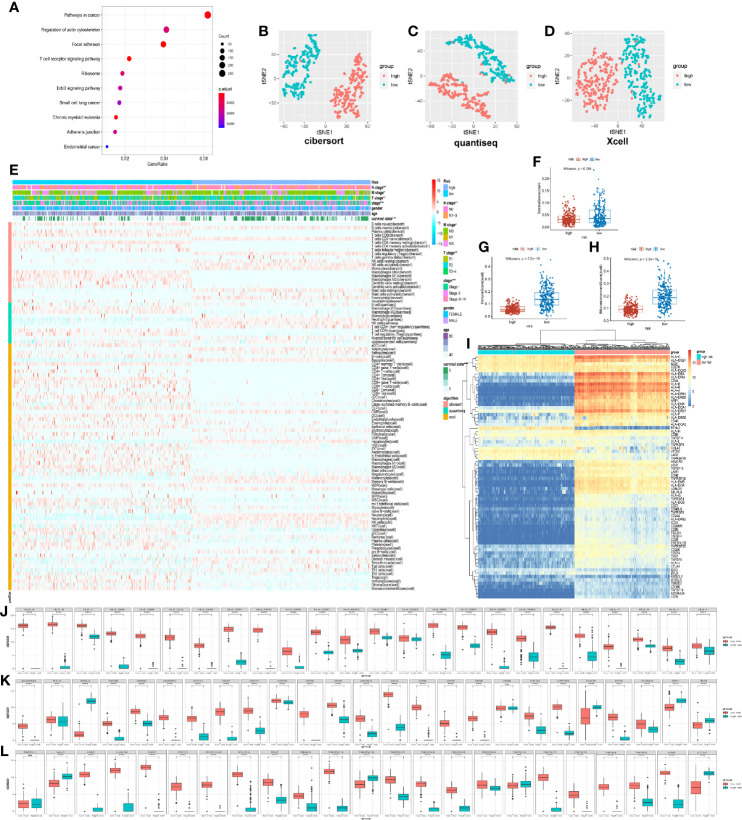
Immune characteristic of prognostic risk model stratification. **(A)** Kyoto Encyclopedia of Genes and Genomes pathway enrichment of different expression genes of high and low risk, demonstrating the top 10. **(B)** t-distributed stochastic neighbor embedding (t-SNE) analysis of 22 immune cells based on CIBERSORT algorithm. **(C)** t-SNE analysis of 11 immune cells based on QUANTISEQ algorithm. **(D)** t-SNE analysis of 64 immune and stroma cells based on XCELL algorithm. **(E)** Heat map demonstrating immune cell infiltration in the high- and low-risk groups in the TCGA databases. The low-risk group had a higher immune infiltration. *P*-values were calculated with chi-square test. The additional annotation of the abscissa included other clinical indexes from TCGA, such as survival state, age, gender, stage, T stage, N stage, M stage, and risk group. The annotation of the vertical axis included three immune infiltrated algorithms, namely, XCELL, QUANTISEQ, and CIBERSORT. **(F–H)** Box plot showing the difference between the high- and low-risk groups about immune score, stroma score, and microenvironment score. **(I)** Heat map demonstrating the difference of human leukocyte antigen (HLA) and immune checkpoint for the high- and low-risk groups. **(J)** Different expression of the HLA gene in the high- and low-risk groups from TCGA. **(K, L)** Different expression of immune checkpoints in the high- and low-risk group from TCGA.

The differentially expressed genes of the high- and low-risk groups were evaluated to research the difference of stratification of the model. By performing KEGG pathway enrichment, these differential genes were discovered to participate in the immune system, such as T cell receptor signal pathways (**Figure 5A**). As was known to us, ubiquitination was a key regulatory mechanism of immune function. As a result, the immune infiltration algorithms of QUANTISEQ (17), CIBERSORT (18), and XCELL (19) studied the relationship between immune cell infiltration of model stratification. Notably, we found out that the immune cells were classified into two distinct groups by the stratification of the model (**Figures 5B–D**). The low-risk group had more infiltration of immune cells, including T cell, B cell, macrophage, and so on (**Figure 4E**). Interestingly, the stratification of models was apparently different in the immune score and environment score, but not the stroma score (**Figures 5F–H**). The low-risk group was higher than the high-risk group in the immune and microenvironment score, which illustrated that the low-risk group was infiltrating with many immune cells. To illuminate the phenomenon, we analyzed the expression associated to immune molecular structure. The human leukocyte antigen (HLA) was coded by major histocompatibility complex (MHC), which was identified by T cell and B cell and tightly associated with immune function, being significantly highly expressed in the low-risk group (**Figure 5I**). Apparently, the expression of HLA was higher in the low-risk group, including HLA-I molecules (HLA-A, HLA-B, and HLA-C) and HLA-II molecules (HLA-DMA, MHC-DMB, and so on) (**Figure 5J**). The expression of immune checkpoint genes (20) in the low-risk group was apparently higher than in the high-risk group, which included T cell and B cell costimulatory molecules (CD28, CD40, and so on) and tumor necrosis factor receptor superfamily (TNFRSF14, TNFR18, and so on) (**Figures 5K, L**). The results demonstrated that the low-risk group infiltrated many immune cells and highly expressed variant immune checkpoints, including programmed death-1 (PD-1), programmed death ligand-1 (PD-L1), and cytotoxic T-lymphocyte antigen-4 (CTLA-4). Accordingly, the immune treatment could benefit these LUAD patients and improve the classified management of patients.

### Patients of the High-Risk Group Showed Higher Mutation and Higher TMB

**Figure 6:**
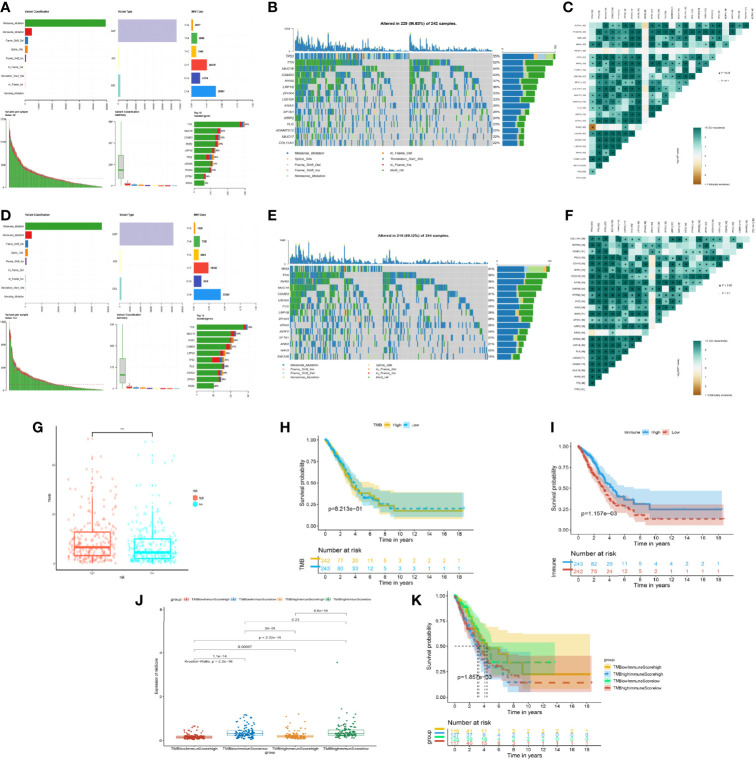
Mutation situation of the prognostic risk model groups. **(A)** Landscape of mutation in the high-risk group from The Cancer Genome Atlas (TCGA). **(B)** Waterfall plot demonstrating the 15 genes with the most mutations and mutated types in the high-risk group from TCGA. **(C)** Co-mutated plots of 25 genes from TCGA by somatic interaction function in the high-risk group from TCGA. **(D)** Landscape of mutation in the low-risk group from TCGA. **(E)** Waterfall plot demonstrating the 15 genes with the most mutations and mutated types in the low-risk group from TCGA. **(F)** Co-mutated plot of 25 genes from TCGA by somatic interaction function in the low-risk group from TCGA. **(G)** Box plot showing the difference between the high- and low-risk groups about tumor mutation burden (TMB). *P*-values were calculated with the Wilcoxon test (***P < 0.001). **(H)** Kaplan–Meier curve of the low- and high-TMB groups. **(I)** Kaplan–Meier curve of the low- and high-immune-score groups. **(J)** Box plot showing the difference of the risk score in the four groups, including high immune score and high TMB, high immune score and low TMB, low immune score and high TMB, and low immune score and low TMB. *P*-values were calculated with the Wilcoxon test. **(K)** Kaplan–Meier curve of four groups.

By the analysis using maftools (21), we found out that mutated tendency and condition were similar in both low- and high-risk groups. The missense mutation of variant classification, SNP of variant type, and SNV class were the same in the stratification of the model. However, the patients’ number of mutated genes in the high-risk group was obviously higher than those in the low-risk model (**Figures 6A, D**). In terms of the top 15 genes of mutation, TP53, TTN, MUC16, CSMD3, RYR2, LRP1B, ZFHX4, USH2A, KRAS, SPTA1, XIRP2, and FLG were the same in the high- and low-risk groups, which showed the importance of the mutation of 12 genes in cancer progression (**Figures 6B, E**). Meanwhile, we analyzed the gene pairs of mutation in the stratification model in terms of Fisher exact examination, visualizing the top 25 gene pairs of mutation (**Figures 6C, F**).

Based on the importance of TMB for immune response treatment in cancer (22–24), the TMB of the high-risk group was higher than that of the low-risk group, which meant that the tumor neoantigen was high in the high-risk group and identified easily by the immune system (**Figure 6G**), whereas the discrepancy of overall survival between the different TMB subgroups was not apparent (**Figure 6H**). Inversely, we found that the OS of the high- and low-immune groups was statistically different (**Figure 6I**). According to the mean of TMB and the immune score of patients, we assigned the patients into four subgroups. Notably, we discovered that the group of low TMB with the high immune score was the lowest in risk score with the best prognosis (**Figures 6J, K**). The phenomenon of the high-TMB group was poor survival and high risk, attributed to the decrease of immune cell response. Hence, integrated TMB and the immune score could benefit the group and improve the health management of patients.

### Drug Response of Clinical Chemotherapy and Target Therapy

**Figure 7:**
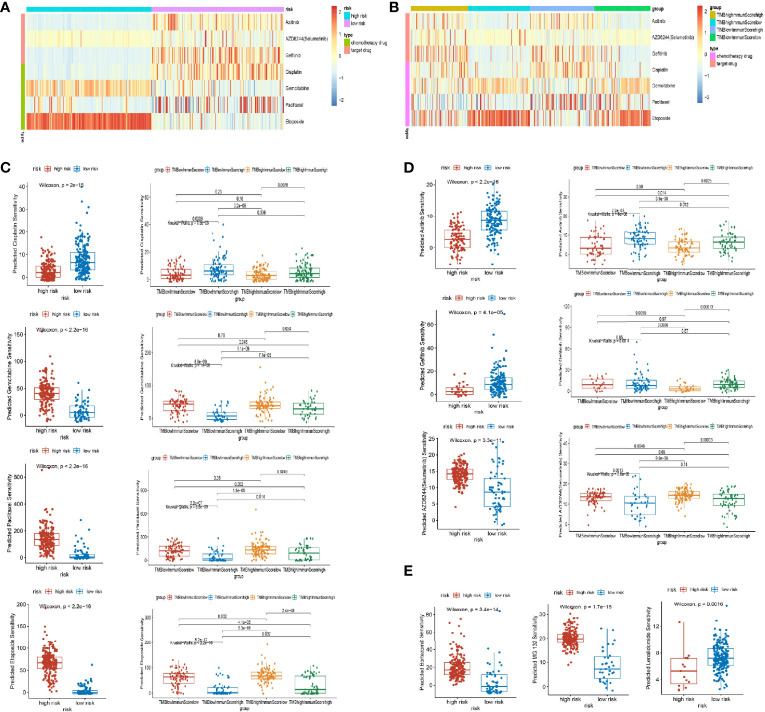
Drug response of subgroups. **(A, B)** Heat map of drug response in the model groups, and groups of tumor mutation burden (TMB) and immune score. **(C)** Different responses of chemotherapy drugs (cisplatin, gemcitabine, paclitaxel, and etoposide) in the model groups and groups of TMB and immune score. **(D)** Different responses of targeted therapy drugs (axitinib, selumetinib, and gefitinib) in the model groups and groups of TMB and immune score. **(E)** Different responses of proteasome inhibitors (bortezomib, MG132, and lenalidomide) in the model groups.

Based on a database of the CGP, we screened 4 chemotherapy drugs (cisplatin, gemcitabine, paclitaxel, and etoposide) and 3 targeted drugs (axitinib, selumetinib, and gefitinib), which had been used in the clinical treatment of LUAD. By analysis of “pRRophetic” package (25), we found out that the drug response was different in the stratification of the ubiquitin model and the group of TMB/immune score (**Figures 7A, B**). In the chemotherapy drug of lung cancer, cisplatin had a high drug response in the low-risk group and the group of high immune score and low TMB. Inversely, gemcitabine, paclitaxel, and etoposide were in high drug responses in the high-risk group and the groups of low immune score and low/high TMB (**Figure 7C**). Additionally, for the targeted drug of lung cancer, axitinib responded highly to the low-risk model and the groups of high immune score and low/high TMB. Gefitinib had a favorable response in the low-risk group, and selumetinib was preferably responded in the high-risk group and the groups of low immune score and low/high TMB (**Figure 7D**). As a consequence, the subgroups of multi-omics could preferably benefit the drug response of patients.

Noticeably, the proteasome inhibitors were implemented in clinical practice, such as bortezomib (proteasome inhibitor), lenalidomide (E3 inhibitors), and MG132 (proteasome inhibitors) (26). In our research, bortezomib and MG132 were favorably responsive in the high-risk group, whereas lenalidomide was highly responsive in the low-risk group of LUAD (**Figure 7E**). As a result, proteasome inhibitors could benefit lung cancer in the future.

## Discussion

Precision treatment brings the gospel for patients bearing advanced LUAD, being largely dependent on comprehensive genomic profiling. Notably, transcriptomic sequencing has been extensively implemented in inpatient wards to facilitate diagnosis and therapeutic regimes, including those being resistant to targeted therapy and even immune checkpoint blockade (27). However, there still exist many challenging unmet clinical needs. Given that several studies have proved that integrated genomic combined with transcriptomic analysis outperforms single-omics analysis (28), how to make much of multi-omics analysis to elaborate on the biological behavior of LUAD and its therapeutic vulnerabilities seems increasingly urgent (29, 30). Here our group presented a ubiquitination-oriented predictive model by means of multi-omics deep profiling. These multilayer molecular architectures of LUAD center on the potential association within ubiquitination and other modifying manners, extensively uncovering its clinical efficacy in detailing biological characteristics and predicting prognosis and drug response to immune checkpoint blockade and targeted therapy.

Severing as a crucial adaptor of protein stability, the ubiquitin–proteasome system is essential for pan-cancer development and process *via* the maintenance of cellular protein homeostasis (31). Ubiquitin recognizes and targets indicated proteins specifically in an enzyme-dependent manner, whose ubiquitination makes themselves vulnerable to degradation. As for now, efforts on ubiquitination are mainly concentrated on a single protein and its upstream or downstream signaling pathways, while the transcriptome, proteomics, and even other multi-omics analyses of ubiquitin signature are minimally in the press. Thus, we took the lead in analyzing the genomes and transcriptome characteristics of enzymes involved in the ubiquitin–proteasome system, expecting to reveal their biological functions and clinical merits in LUAD.

In our study, based on LASSO regression analysis of transcriptome data, 9 core genes of ubiquitin were screened to eventually construct the prognosis risk model, including USP29, MPP7, TRIM40, HERC1, TLE1, ASB2, NEDD1, USP44, and PHF1, which would be the new prognostic biomarkers in LUAD. Noticeably, some of them have been validated to share a close connection with LUAD, while the others seem undervalued. Previous research proved that USP29 upregulation enhances the cancer stem cell-like characteristics in lung adenocarcinoma cells to promote tumorigenesis in athymic nude mice (32). It was also found that, within a human lung tumor tissue array, a significant number of carcinomas overexpress TLE1 and correlate with malignancy in cancer, regarded as a biomarker to predict the prognosis of LUAD patients (33, 34). Meanwhile, USP44 is frequently downregulated in lung cancer, leading to a poor prognosis, which is further corroborated in mice (35). Although other genes have seldom been reported, it is worthy to explore their potential relationship with carcinogenesis and the proteomics landscape in LUAD.

According to the nomogram, a steady and credible tool to quantitatively measure the risk on an individual basis by combining and delineating the risk factors (36), our study demonstrated that the model of ubiquitin signature is tightly associated with the risk and prognosis of patients. In the high-score cohort, the survival time and survival quantity were significantly lessened, which is superior to other conventional predictive methods due to its independence in prediction. An integrated model combined with the TNM stage may be utilized to comprehensively predict the risk and prognosis of clinical patients in clinical practice in order to acquire favorable clinical management. Furthermore, this model also prompts clinical traits and the progress of LUAD. The phenotype-related stratification of the model assigned the patients into two distinct subgroups. In the low-score cohort, the overall survival of patients overweighted those with high scores in the early or advanced stages, respectively. These results indicated that the integrated utilization of a ubiquitination-oriented model and other clinical indexes may potentially optimize the clinical management of LUADs.

We also found that this model can be applied to predict representative immune checkpoint inhibitor responses to LUAD *via* innate and adaptive immunity, respectively (10, 37). It was reported that RIG-I-like receptor and neuron-derived neurotrophic factors might awaken lung cancer by the immune system (14–16). In this study, by the PPI and GSEA analysis of each gene within this model, 9 genes referred to RIG-I-like receptor, neuron-derived neurotrophic factors, cancer, and ubiquitin–proteasome system in lung adenocarcinoma, illuminatingly manifesting that the ubiquitin–proteasome system has a potential association with the immune system in cancer. Moreover, ubiquitin also functions in T cell-mediated adaptive immune responses. By the t-SNE analysis of immune infiltration of QUANTISEQ, XCELL, and CIBERSORT algorithms, we separated the engaged populations into two specific subgroups, namely, high- and low-score cohorts. The clinical samples in the low-score group were infiltrated with diverse immune cells, including B cells, T cells, and DC, and possessed escalated expressions of HLA-I and HLA-II. Analogously, the costimulatory molecules, TNFR superfamily, and microenvironment score are consistent with the ascendant expression of immune checkpoint markers in this cohort (38, 39). On account of PD-1, PD-L1, and CTLA-4 highly expressed in the low-risk model, the indicated cohort is susceptible to immune checkpoint blockade, theoretically facilitating the clinical selection of the beneficial population. TMB facilitates the establishment of personalized immunotherapy approaches within genomic sequencing among LUAD patients, which has been accepted as an independent predicting factor to immune checkpoint inhibitors (40, 41). Nevertheless, the discrepancy in overall survival between the different TMB subgroups is not apparent in our research. We found that those with high TMB suffered a higher risk and a more undesirable prognosis than those in the opposite group (41). Noticeably, integrated TMB and the immune score could address this dilemma. Our observations indicated that those with high TMB and low immune scores were subjected to a high risk of development and weakened survival rate. Inversely, survival and prognosis are superior for those of the three other groups in the group of low TMB and high immune scores. These outcomes demonstrated that the performance of integrating TMB and immune score outperforms the single TMB in the prognosis of LUAD patients.

Platinum-based chemotherapy regime and targeted therapy improve the patient’s survival rate of LUAD to an extent (42, 43). Cisplatin, gemcitabine, paclitaxel, and etoposide give rise to the benefit of LUADs and inhibit the progress of lung cancer (44–48), and the personalization of targeted therapy to corresponding markers (axitinib, selumetinib, and gefitinib) also contributes to the extension of life expectancy (49–51). According to the above-mentioned details, we found that gemcitabine, paclitaxel, etoposide, and selumetinib presented differential responses to the two stratifications of this multi-omics model, showing a high response in the high-risk group and a boost of curative effects, but for cisplatin, axitinib, and gefitinib, they seem to benefit those in the low-risk cohort. Analogously, the groups of genome alternation demonstrate the different drug responses of subgroups. Drawing from these results, we can conclude that sophisticated stratification can further uncover the application value of ubiquitin-related multi-omics profiling, in turn advancing the pertinent individualized therapy scheme in the clinic.

Unfortunately, there are several limitations needed to be recognized in this study. To start with, the sample size was inadequate to reflect objective facts in the real world. Due to the orientation from limited public databases, the samples were restricted to a confined population that we could not analyze additional detailed information, which might be consistent with reality. Furthermore, restricted sample sequencing failed to optimize the potential clinical application value of this ubiquitination model. This predictive model was drawn from sequence profiling to specific populations in databases and, in turn, failed to verify its specificity and sensitivity in clinical prediction. High-throughput sequencing combined with multi-omics of lung adenocarcinoma tissues endows this model increased practical merits, which costs a high expense and will be engaged in our coming research scheme. Finally, rough risk factor stratification weakened its predictive efficiency of the indicated layers to drug responses. Taking defined non-quantitative scores as a distinctive criterion for evaluating drug responses cannot meet the demand for discrimination and precise therapeutical regimes. Combined standards with TMB or other indicating markers is still preferred to a cursory single one, which needs further exploration in the near future.

## Conclusion

In summary, our study revealed the clinical application value of a ubiquitination-oriented predictive model from public databases. Integrated and stratified multi-omics analyses within immune infiltration and genome alternation are conducive to illustrate its clinical potency in describing ubiquitin characteristics, escalating precision therapy, and predicting prognosis.

## Data Availability Statement

The datasets presented in this study can be found in online repositories. The names of the repository/repositories and accession number(s) can be found in the article/**Supplementary Material**.

## Author Contributions

YC, DJ, YZ, JZ, and KY conceptualized and designed the study. YC, DJ, and YZ took charge of methodological development. YC, DJ, JF, HX, YZ, YS, YW, and LX took charge of the acquisition of data. YC, YZ, YS, JP, and NC collected and analyzed the data. YC, YZ, DJ, KY, JZ, YW, and LX wrote, reviewed, and revised the manuscript. JZ and KY supervised the study. All authors contributed to the article and approved the submitted version.

## Funding

The cost of this work was funded by the National Natural Science Foundation of China (no. 81902316 to YZ, no. 81773153 to JZ, and no. 82073154 to KY).

## Conflict of Interest

The authors declare that the research was conducted in the absence of any commercial or financial relationships that could be construed as a potential conflict of interest.

## Publisher’s Note

All claims expressed in this article are solely those of the authors and do not necessarily represent those of their affiliated organizations, or those of the publisher, the editors and the reviewers. Any product that may be evaluated in this article, or claim that may be made by its manufacturer, is not guaranteed or endorsed by the publisher.

## Supplementary Material

Supplementary Figure S1 Comparing three models. **(A, C, E)** The Kaplan-Meier curve of the high and low-risk groups by constructing the URS_multi-cox_ model, URS_LASSO (11)_ model, and URS_LASSO (9)_ model. **(B, D, F)** The average 1-year, 3-year and 5-year ROC curve of the risk score by constructing the URS_multi-cox_ model, URS_LASSO (11)_ model, and URS_LASSO (9)_ model.

Supplementary Figure S2 The Kaplan-Meier curve of overall survival of LUAD patients. **(A–I)** The Kaplan-Meier curve of overall survival of LUAD patients in each gene of URSLASSO (9) model, including ASB2, HERC1(p619), MMP7, NEDD1, PHF1, TLE1, TRIM40, USP29, and USP44.

Supplementary Figure S3 The Kaplan-Meier curve of first progression of LUAD patients. **(A–I)** The Kaplan-Meier curve of first progression of LUAD patients in each gene of URSLASSO (9) model, including ASB2, HERC1(p619), MMP7, NEDD1, PHF1, TLE1, TRIM40, USP29, and USP44.

## Abbreviations

LUAD, lung adenocarcinoma; GEO, Gene Expression Omnibus; TCGA, The Cancer Genome Atlas; GO, Gene Ontology; KEGG, Kyoto Encyclopedia of Genes and Genomes; t-SNE, t-distributed stochastic neighbor embedding; TMB, tumor mutation burden; CGP, Cancer Genome Project; ROC, receiver operating characteristic curve; HLA, human leukocyte antigen; MHC, major histocompatibility complex
