# Supplementary figures and images for: The Clinical Prediction Value of the Ubiquitination Model Reflecting the Immune Traits in LUAD
Source: Front Immunol. 2022 Feb 25;13:846402. doi: 10.3389/fimmu.2022.846402 (PMC8913715; doi:10.3389/fimmu.2022.846402)

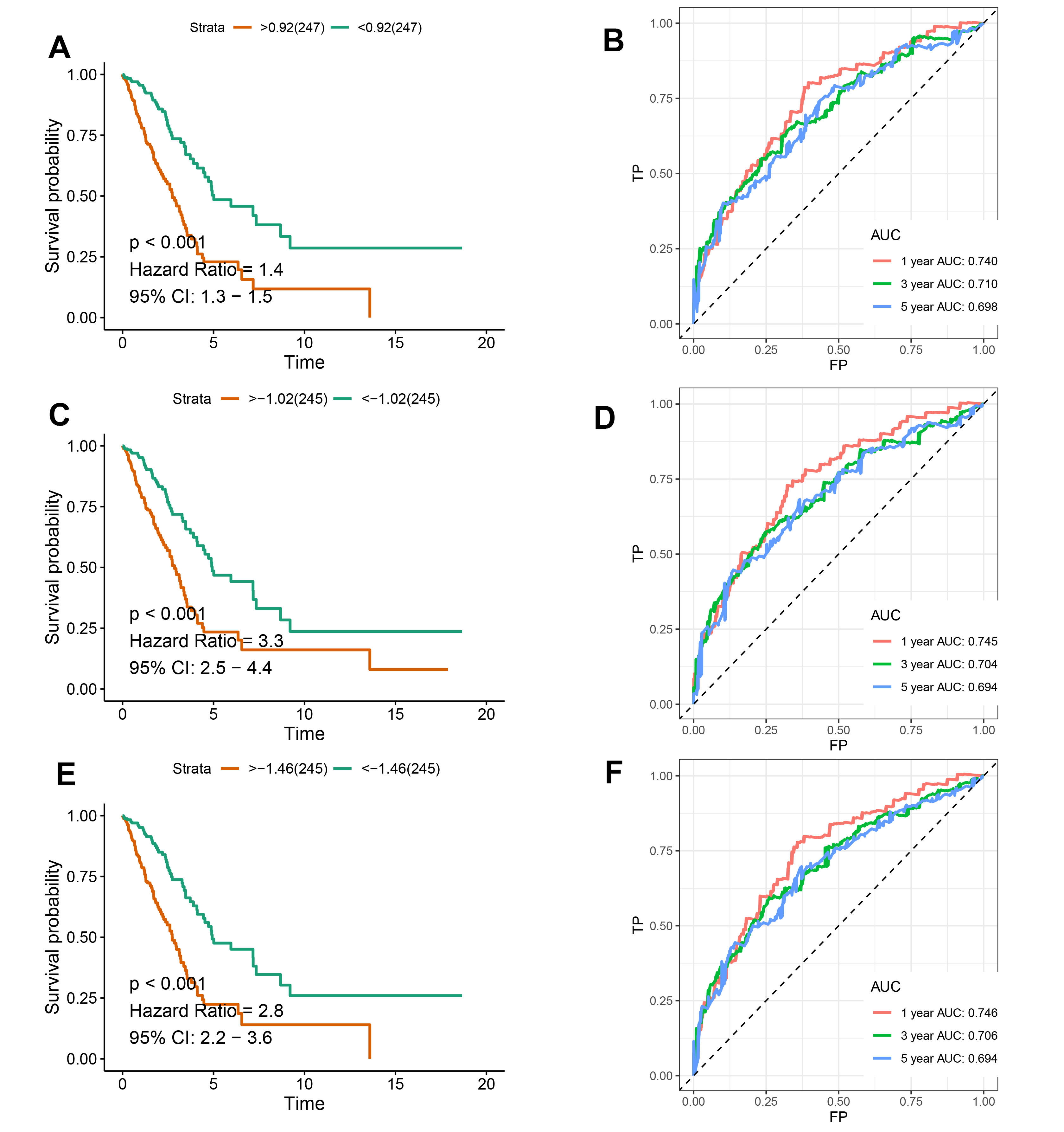

Supplement: Supplementary Figure S1 — Comparing three models. (A, C, E) The Kaplan-Meier curve of the high and low-risk groups by constructing the URSmulti-cox model, URSLASSO (11) model, and URSLASSO (9) model. (B, D, F) The average 1-year, 3-year and 5-year ROC curve of the risk score by constructing the URSmulti-cox model, URSLASSO (11) model, and URSLASSO (9) model. [file Image_1.tif]

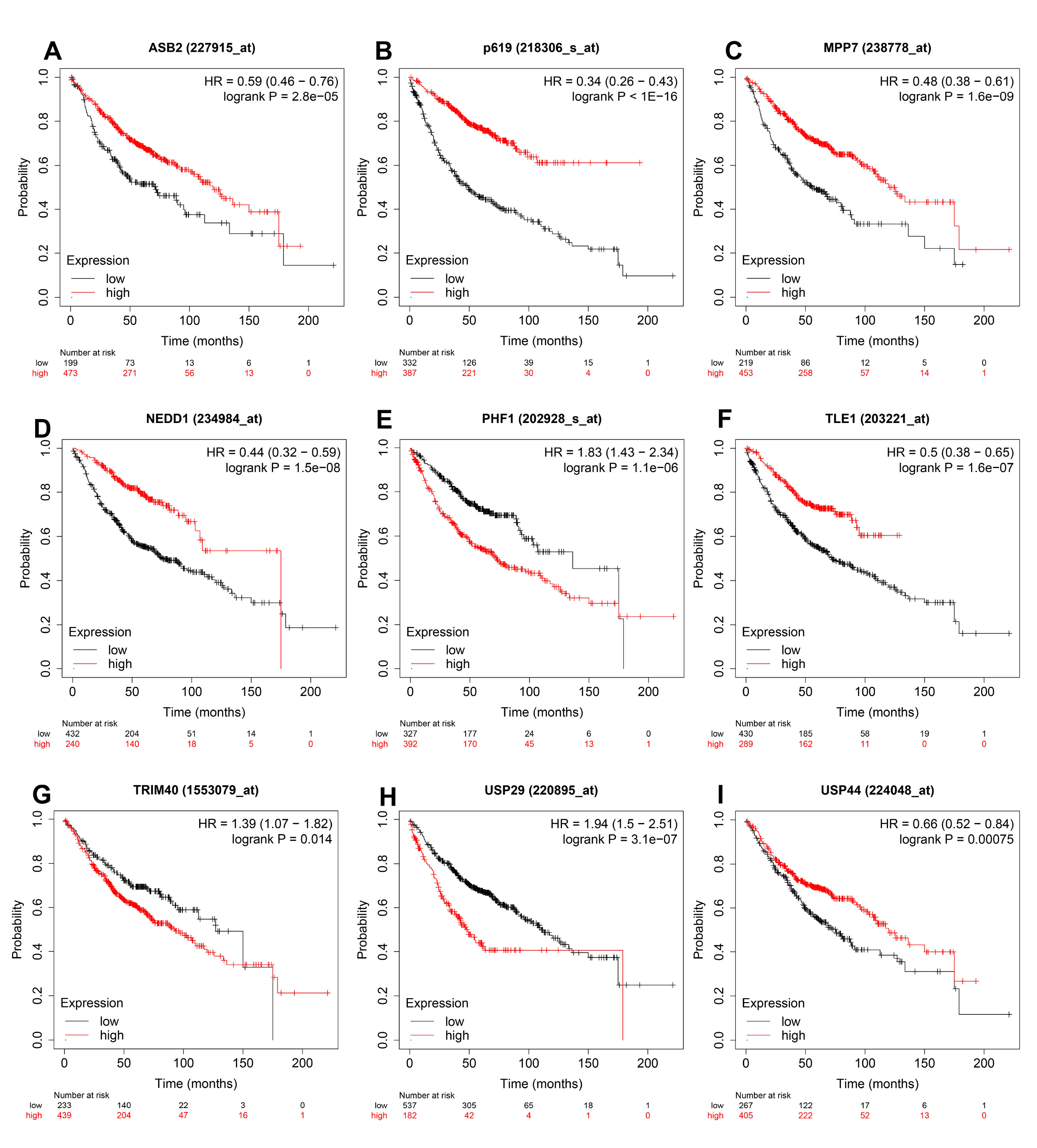

Supplement: Supplementary Figure S2 — The Kaplan-Meier curve of overall survival of LUAD patients. (A–I) The Kaplan-Meier curve of overall survival of LUAD patients in each gene of URSLASSO (9) model, including ASB2, HERC1(p619), MMP7, NEDD1, PHF1, TLE1, TRIM40, USP29, and USP44. [file Image_2.tif]

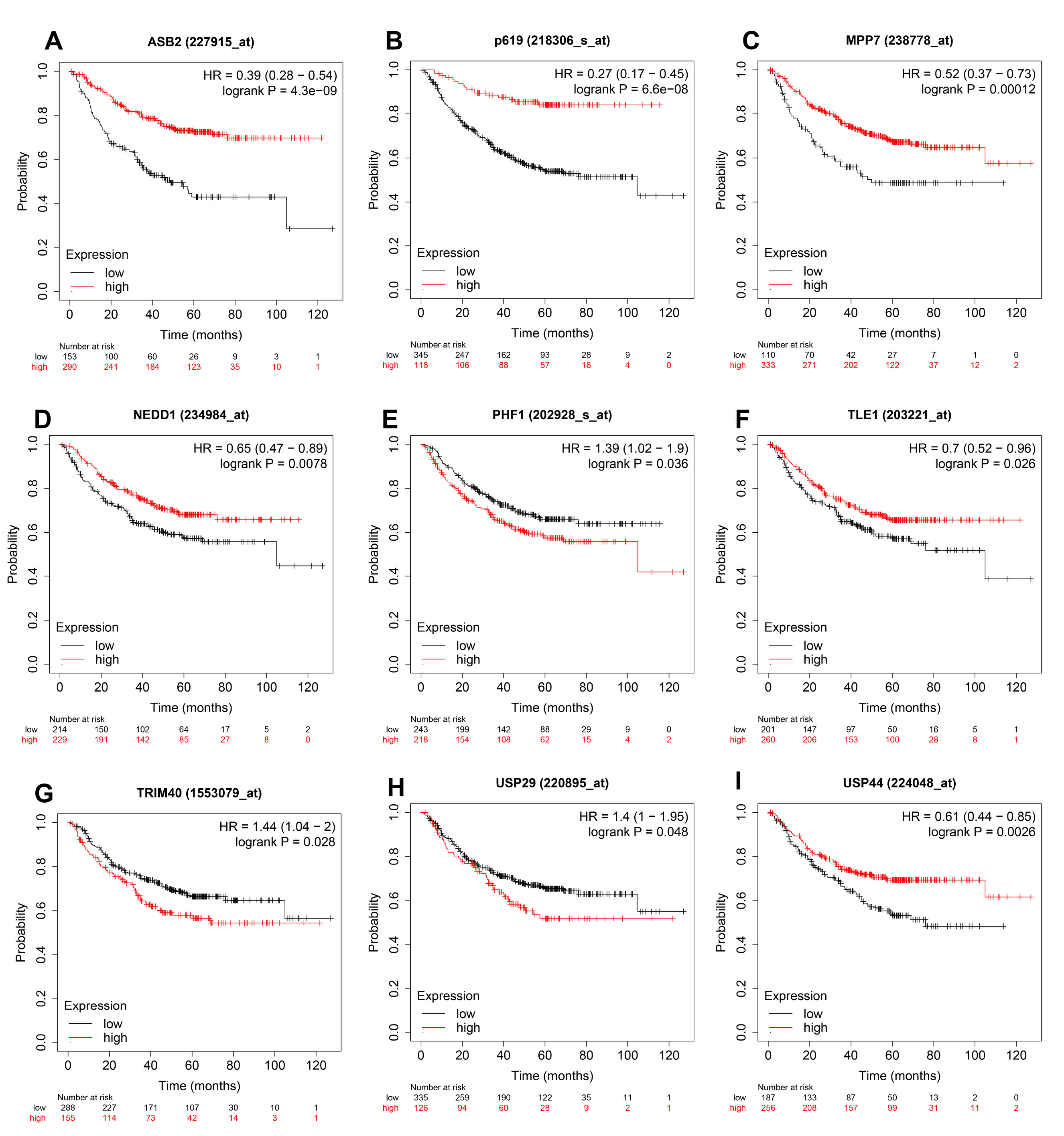

Supplement: Supplementary Figure S3 — The Kaplan-Meier curve of first progression of LUAD patients. (A–I) The Kaplan-Meier curve of first progression of LUAD patients in each gene of URSLASSO (9) model, including ASB2, HERC1(p619), MMP7, NEDD1, PHF1, TLE1, TRIM40, USP29, and USP44. [file Image_3.tif]
